# Understanding adolescent stress and coping through psychodynamic constructs: evidence from a comparative study

**DOI:** 10.3389/fpsyg.2025.1668051

**Published:** 2025-09-23

**Authors:** Aslı Akın, Kirstin Goth, Inge Seiffge-Krenke, Lea Sarrar

**Affiliations:** ^1^Department of Psychiatry and Psychotherapy, Charité-Universitätsmedizin Berlin, Berlin, Germany; ^2^Department of Psychology, Faculty of Sciences, MSB Medical School Berlin, Berlin, Germany; ^3^Department of Child and Adolescents Psychiatry, University Clinics Saarland, Homburg, Germany; ^4^Department of Psychology, University of Mainz, Mainz, Germany

**Keywords:** adolescence, stress perception, coping behavior, psychodynamic conflicts, personality structure, defense style

## Abstract

**Introduction:**

Adolescence is a critical developmental phase marked by increased vulnerability to stress and the formation of functional or dysfunctional coping strategies. While stress and coping are well-studied for their psychopathological relevance, their associations with psychodynamic constructs—such as impairments in personality structure, psychodynamic conflicts, and defense mechanisms-remain underexplored. This study investigated whether these psychodynamic features are associated with stress perception and coping styles among adolescents with and without mental health problems.

**Methods:**

A total of 171 adolescents (aged 14–21) completed standardized self-report measures assessing stress across multiple life domains, coping styles, psychodynamic conflicts, impairments in personality structure, and habitual defense styles. Mental health problems were screened via self-reports, and participants were classified into clinical and healthy control groups accordingly.

**Results:**

Adolescents with mental health problems reported significantly higher stress and more dysfunctional (avoidant) coping strategies than their healthy peers. Regression analyses revealed that impairments in personality structure—particularly in identity and attachment—predicted heightened stress perception and dysfunctional coping, especially in the clinical group. Psychodynamic conflicts showed domain-specific links to stress: the guilt conflict was associated with school- and family-related stress, while the passive oedipal conflict predicted stress in romantic relationships. Maladaptive defense style was negatively associated with self- and peer-related stress, suggesting dissociative and affect-isolating mechanisms limiting conscious stress perception. Among healthy adolescents, functional (active) coping was associated with fewer impairments in attachment.

**Discussion:**

Findings highlight the value of psychodynamic constructs for understanding adolescent stress and coping and the relevance of attachment- and personality structure-focused prevention and intervention strategies.

## Introduction

Adolescence is a critical developmental period characterized by intense biological, psychological, and social transformation. During this phase, familiar routines and behavioral patterns lose their stability, and adolescents are confronted with a variety of age-specific developmental tasks—such as detaching from the parental home, forming new social bonds, integrating bodily changes into their self-concept, exploring sexual identity, or making career-related decisions. These challenges necessitate the acquisition, adaptation, and refinement of individual coping strategies, which are closely linked to how stress is perceived and regulated (Stelzig and Sevecke, 2019).

According to Lazarus and Folkman's transactional stress model (1984), stress arises from an imbalance between perceived demands and personal resources. Coping strategies—whether active (e. g., problem-solving, support seeking), internal (e. g., cognitive processing, considering possible solutions), or avoidant (e. g., withdrawal, denial)—play a critical role in how adolescents manage stress (Seiffge-Krenke, 1993, 1995). While active and internal coping are considered functional and solution-oriented, avoidant coping is seen as dysfunctional due to its evasive nature (Beyer and Lohaus, 2007; Gelhaar et al., 2007).

Cross-cultural research has shown that adolescents are particularly affected by stress related to future uncertainties (Seiffge-Krenke et al., 2012). As they move through adolescence, their overall stress perception tends to rise—especially in academic and social contexts (Seiffge-Krenke et al., 2009). At the same time, adolescents gradually shift from passive to more active and differentiated coping strategies (Seiffge-Krenke et al., 2009). A growing body of research further highlights the mediating role of coping in the link between stress exposure and mental health outcomes during this critical developmental stage (Meng et al., 2011; Seiffge-Krenke, 2000; Su et al., 2022). Longitudinal findings also suggest gendered developmental pathways, with functional coping during adolescence predicting lower symptomatology in young adult women (Seiffge-Krenke and Persike, 2017).

The prevalence and psychopathological relevance of perceived stress and specific coping strategies during adolescence underscore the clinical importance of studies investigating predictors that influence the development and utilization of functional coping. Interpersonal factors such as perceived parental autonomy support have been shown to shape adolescents' coping development (Seiffge-Krenke and Pakalniskiene, 2011). Regarding intrapersonal factors, playfulness may reduce perceived stress but appears unrelated to coping style (Staempfli, 2007). Longitudinal findings link secure attachment models to lower relational stress and more active coping (Allen et al., 2004; Seiffge-Krenke, 2006). Additionally, an international study found that neurotic personality traits are associated with identity stress and dysfunctional coping in somatically burdened young adults (Seiffge-Krenke and Sattel, 2024).

Despite broad cross-cultural research on stress and coping in adolescence, psychodynamic perspectives remain largely absent from this field, although they are central to clinical diagnostics and psychotherapy. The psychodynamic approach emphasizes unconscious intrapsychic processes—such as inner conflicts, personality structure, and defense mechanisms—which fundamentally shape adolescents' emotional experiences and behavioral tendencies, and therefore also influence how they perceive, interpret, and manage stress.

Previous research indicates that especially psychodynamic conflicts—defined as enduring tensions between opposing motivational forces within a person (Task Force OPD-CA-2 (Ed.), 2020)—are ubiquitous during adolescence (e.g., Akın et al., 2021; Escher et al., 2021). Within the Operationalized Psychodynamic Diagnosis in Childhood and Adolescence framework (OPD-CA; Task Force OPD-CA-2 (Ed.), 2020), seven core psychodynamic conflicts can be distinguished: closeness vs. distance, submission vs. control, taking care of oneself vs. being cared for, self-worth, guilt, oedipal, and identity conflict. These conflicts are assumed to be shaped by early childhood relationship experiences and to be reactivated during adolescence, when new relational challenges arise as adolescents increasingly detach from their parents (Blos, 1962). When strongly pronounced, however, these conflicts are linked to a range of psychopathology (e.g. Bagattini, 2021). Beyond conflicts, psychodynamic theory and research highlights impairments in personality structure—stable patterns that govern emotional experience, identity, interpersonal functioning, and self-regulation (Task Force OPD-CA-2 (Ed.), 2020). For example, during the COVID-19 pandemic—a global *stressor*—various mental health problems in adolescents were significantly associated with impairments in both overall personality structure and specific sub-domains, including attachment, identity, interpersonality, and control (Akın and Sarrar, 2024). Another core concept in psychodynamic research and practice is defense mechanisms, which reflect unconscious responses to internal or external stressors (Task Force OPD-CA-2 (Ed.), 2020). Defense mechanisms can be categorized by maturity level, with immature defense styles—such as projection or splitting—being associated with personality pathology (Laczkovics et al., 2025), anxiety and depression (Ferrajão et al., 2024), as well as internalizing and externalizing problem behavior in adolescence (Huemer et al., 2015).

However, studies systematically connecting psychodynamic constructs with stress perception and coping strategies in adolescents remain scarce. This study seeks to address this gap by integrating OPD-CA-based concepts—psychodynamic conflicts, personality structure, and defense styles—into contemporary stress and coping research. By doing so, we aim to move beyond descriptive accounts of stress in adolescence and provide a theoretically grounded framework that links unconscious processes with observable coping behavior.

Using a controlled study design, we investigated associations between subjective stress experience, coping behavior, and psychodynamic features in two groups of adolescents: those with mental health problems and those without. We hypothesized that, among adolescents with mental health problems, higher levels of psychodynamic conflicts, structural impairments, and an immature defense style would be associated with greater stress perception and dysfunctional (avoidant) coping. In contrast, among adolescents without mental health problems, functional coping was expected to be linked to a mature defense style, lower levels of psychodynamic conflicts, and fewer structural impairments.

In clarifying these associations, our study offers two contributions. First, it expands stress–coping research by systematically including psychodynamic constructs, which have so far been neglected despite their clinical relevance. Second, it highlights unique clinical implications: a psychodynamic understanding of stress and coping may guide individualized interventions by linking adolescents' coping behavior to underlying conflict constellations, structural vulnerabilities, and defense styles. Such knowledge may support clinicians in tailoring preventive and therapeutic strategies more precisely to adolescents' developmental and intrapsychic profiles.

## Materials and methods

### Study design and sample

The present study is a cross-sectional investigation conducted in Germany. It is part of a larger research project at MSB Medical School Berlin that incorporates both cross-sectional and longitudinal approaches to examine psychodynamic and psychopathological characteristics in intrafamilial contexts through self-assessment tools. In the overall project, data from *N* = 906 adolescents, their *N* = 426 mothers, and *N* = 262 fathers have now been collected.

A total of *n* = 171 (*M*_*age*_ = 18.0; *SD* = 2.3; 61% female and 39% male sex) adolescents between the ages of 14 and 21 years, who provided complete standardized information on their stress experience, coping behavior, and psychodynamic characteristics, were included in this study. Of these, *n* = 84 participants (*M*_*age*_ = 18.4; *SD* = 2.1; 67% female and 33% male sex) were identified as experiencing mental health problems, as assessed by the German version of the *Patient Health Questionnaire* (PHQ-D, Löwe et al., 2002), while *n* = 87 adolescents (*M*_*age*_ = 17.7; *SD* = 2.3; 54% female and 46% male sex) formed a healthy control group, in which mental health problems were ruled out based on PHQ-D (Löwe et al., 2002) results. Within the clinical sample (*n* = 84), 22 participants (26%) met criteria for a somatoform syndrome, 23 (27%) for a depressive syndrome (including 2 [2%] mild, 22 [26%] moderate, and 14 [17%] severe cases), 13 (16%) for panic disorder, 13 (16%) for another anxiety disorder, 4 (5%) for bulimia nervosa, 6 (7%) for binge eating disorder, and 41 (49%) for harmful alcohol use. In terms of socioeconomic status (SES), adolescents with mental health problems were distributed as follows: 49% came from high SES backgrounds, 29% from middle SES, 19% from low SES, and 3% from very low SES backgrounds. In comparison, within the mentally healthy control group, 63% had a high SES, 20% a middle SES, 13% a low SES, and 4% a very low SES.

Participants were recruited through secondary schools and youth centers in Germany. Both clinical and control group participants were approached through the same recruitment sources to reduce systematic bias (see Supplementary Table S1 for a detailed breakdown of recruitment sources by group). Written informed consent was obtained from all participants in accordance with the principles outlined in the Declaration of Helsinki. For participants under the age of 18, additional written consent was obtained from a parent or legal guardian. The study adhered to applicable data protection regulations and ethical standards. The study protocol was approved by the ethics committee of MSB Medical School Berlin (approval number: MSB-2020/30).

### Measures

#### Perceived stress

Adolescents' perceived stress was assessed using the *Problem Questionnaire* (PQ; Seiffge-Krenke, 1995), which captures everyday stressors that are commonly encountered during adolescence across various life domains. The instrument comprises 64 items reflecting typical minor stressors identified in prior research. Participants rated how stressful each situation was for them on a 5-point Likert scale ranging from 1 (“not stressful at all”) to 5 (“highly stressful”). Based on previous factor-analytic findings (Seiffge-Krenke, 1995), the PQ encompasses seven stress domains: school (e.g., Item 1: “There is great pressure to get the best marks in school”), future (e.g., Item 14: “I don't know what I'm going to do when I leave school.”), family (e.g., Item 22: “I can't talk with my parents”), peers (e.g., Item 33: “I'm unsure whether the others will accept me”), leisure time (e.g., Item 38: “School and domestic commitments leave me with too little free time and I don't know what to do after I leave school.”), romantic relationships (e.g., Item 46: “I'm afraid of losing contact with my friends if I pair up with a boyfriend/girlfriend”), and self (e.g., Item 58: “I am dissatisfied with my behavior, qualities and skills.”). In the present study, internal consistencies of the subscales were acceptable to very good, with McDonald's Omega values ranging from 0.66 to 0.88: school (ω = 0.77), future (ω = 0.71), family (ω = 0.86), peers (ω = 0.82), leisure time (ω = 0.66), romantic relationships (ω = 0.79), and self (ω = 0.88). These values are comparable to those reported by (Seiffge-Krenke 1995), who found Cronbach's alpha coefficients ranging from 0.70 to 0.84 across the seven domains.

#### Coping strategies

To assess adolescents' coping strategies, the *Coping Across Situations Questionnaire* (CASQ; Seiffge-Krenke, 1995) was administered. The CASQ measures 20 distinct coping strategies across eight potential problem domains during youth: school, teachers, family, peers, romantic relationships, self, leisure time, and future. Participants were asked to indicate which coping strategies they typically use when facing a problem in each of the eight applicable domains. Multiple strategies could be selected per domain, and each strategy could be endorsed up to eight times—once per domain. A factor analysis conducted by (Seiffge-Krenke 1995) identified three higher-order coping styles: active coping (6 items; e.g., Item 1: “I discuss the problem with my parents”), internal coping (7 items; e.g., Item 10: “I think about the problem and try to find different solutions”), and withdrawal (7 items; e.g., Item 20: “I withdraw, because I cannot change anything anyway”). In the present study, scores for each coping style were computed as sum scores across all items and domains of the CASQ. The internal consistency of the dichotomous scale was assessed using the Kuder–Richardson Formula 20 (KR-20), which is equivalent to Cronbach's alpha for binary items. Reliability coefficients were good to excellent with 0.89 for active coping, 0.89 for internal coping, and 0.90 for withdrawal. These values are consistent with previous findings reported by (Seiffge-Krenke 1995), who found Cronbach's alpha coefficients ranging from 0.73 to 0.88 across the three coping dimensions.

#### Psychodynamic conflicts

Psychodynamic conflicts were assessed using the *OPD Conflict Questionnaire for Adolescents* (OPD-CA-CQ; Seiffge-Krenke and Escher, 2021). This self-report instrument measures the seven psychodynamic conflicts defined in the OPD-CA system (Task Force OPD-CA-2 (Ed.), 2020) and distinguishes between active and passive modes of conflict processing. The questionnaire includes 28 items, each rated on a five-point Likert scale ranging from 0 (“no”) to 4 (“yes”). Each conflict is assessed with four items, two measuring the active mode and two measuring the passive mode of conflict processing. Higher mean scores on the respective conflict dimensions reflect more pronounced manifestations of the underlying psychodynamic conflicts. Example items include: „I prefer to be alone, I can't rely on others“ (closeness vs. distance conflict in active mode), „I want to be admired“ (self-worth conflict in active mode), „I blame myself when something goes wrong in my family“ (guilt conflict in passive mode) or „I am quite inhibited when dealing with the opposite sex“ (oedipal conflict in passive mode). In the present study, internal consistencies of the 14 conflict scales (each consisting of two items) were estimated using the Spearman–Brown coefficient. All coefficients were above 0.99, indicating extremely high reliability. However, due to the small number of items and the unusually high inter-item correlations, these values should be interpreted with caution. The questionnaire has demonstrated satisfactory to good reliability values for all scales according to (Seiffge-Krenke and Escher 2021). In contrast, other studies have found low reliability values for some conflict scales (Cropp and Claaßen, 2021; Weber et al., 2020).

#### Personality structure

Adolescents' personality structure was assessed using the *OPD-CA2-Structure Questionnaire* (OPD-CA2-SQ; Goth et al., 2018), a self-report instrument based on the OPD-CA (Task Force OPD-CA-2 (Ed.), 2020). The questionnaire captures impairments across key domains of personality functioning relevant to psychodynamic theory, including identity, attachment, interpersonality, and control. It comprises 81 items rated on a 5-point Likert scale ranging from 0 (“no”) to 4 (“yes”). Higher scores indicate more pronounced impairments in personality structure. For example, the item “I often don't know who I really am” reflects disturbances in the key domain identity, while “When I am alone I often get very scared” refers to impairments in the key domain attachment. Items such as “I often don't understand other people's reactions to my behavior” assess the key domain interpersonality, and “Sometimes I'm so angry that I can't guarantee anything” captures difficulties in the key domain control. In the present study, internal consistencies (McDonald's ω) were acceptable to excellent: ω = 0.91 for identity, ω = 0.86 for attachment, ω = 0.74 for interpersonality, and ω = 0.90 for control. Factor-analytic and validation studies have supported the questionnaire's structural validity and reliability in clinical and non-clinical adolescent populations (Schrobildgen et al., 2019).

#### Defense style

Adolescents' habitual defense mechanisms were assessed using the *Defense Style Questionnaire for Adolescents* (DSQ-22-A; Sarrar and Goth, 2022), a self-report instrument adapted for adolescents based on the 40 item version of the Defense Style Questionnaire for adults (DSQ-40; Andrews et al., 1993). The DSQ-22-A comprises 22 items rated on a 9-point Likert scale ranging from 0 (“not true”) to 8 (“completely true”). Each item captures a characteristic defense-related attitude or behavior, and the instrument differentiates between three empirically and theoretically derived defense styles: adaptive [e.g., sublimation: “I get rid of bad feelings like sadness or anxiety by doing something creative or meaningful (like painting, sports, music)”], neurotic (e.g., idealization: “I have often had the feeling that someone I know is like a guardian angel”), and maladaptive defense style (e.g., splitting: “Sometimes I think I am good like an angel and other times I think I am bad and evil like a devil”). Higher scores on each factor reflect a stronger use of the respective defense style. In the present study, internal consistencies (McDonald's ω) were acceptable for the adaptive (ω = 0.69) and maladaptive defense style (ω = 0.71), while reliability for the neurotic defense style was low (ω = 0.50). (Sarrar and Goth 2022) reported acceptable scale reliabilities for all three defense styles in a clinical and school sample containing 396 adolescents.

#### Mental health problems

The PHQ-D (Löwe et al., 2002) was used as a self-report screening tool to assess mental health problems. It comprises 58 items covering a broad range of syndromal-level disorders, including somatoform, depressive, anxiety, eating, and alcohol-related disorders. Items are answered on dichotomous as well as three- to four-point Likert scales, depending on the diagnostic domain. In this study, the somatoform syndrome scale (McDonald's ω = 0.75) and the depressive syndrome scale (McDonald's ω = 0.83) demonstrated acceptable to good internal consistencies. Previous psychometric analyses of the PHQ-D have indicated excellent reliability across its core scales (Gräfe et al., 2004). For the remaining diagnostic domains, internal consistency coefficients were not calculated, as these subscales are primarily interpreted categorically based on established jump rules.

### Data analysis

To examine whether adolescents with and without mental health problems differ in their perceived stress and coping behavior, multivariate analysis of variance (MANOVA) were conducted. In a first MANOVA, the independent variable was mental health status (based on the PHQ-D), and the dependent variables were the seven PQ stress domains. The second MANOVA examined group differences in coping behavior, with mental health status again as the independent variable and active coping, internalizing coping, and withdrawal as dependent variables. Prior to analysis, the assumptions of multivariate normality, linearity, homogeneity of variances and covariances (Box's M test), and absence of multicollinearity were examined, with no severe violations detected.

In a second step, a series of multiple linear regression analyses was conducted to investigate the extent to which adolescents' perceived stress and coping behaviors can be explained by psychodynamic characteristics, including impairments in personality structure, defense styles, and psychodynamic conflicts. Separate regression models were estimated for adolescents with and without mental health problems to explore potential group-specific patterns in the predictive value of psychodynamic variables. To account for potential confounding effects, age, sex, and SES were entered directly into all models as covariates, while the psychodynamic predictors were selected using a stepwise method. The stepwise method was chosen due to the exploratory nature of the study, allowing identification of the most relevant psychodynamic predictors from a larger set of variables in the absence of strong a priori hypotheses. In addition to the stepwise regression models reported here, we conducted robustness checks using alternative entry methods (e.g., forced entry of conceptually relevant predictors and exclusion of certain predictors; see Supplementary Table S2). The resulting patterns of predictors were highly consistent with the stepwise models. All regression assumptions (linearity, normal distribution of residuals, homoscedasticity, independence of errors, and multicollinearity) were checked using standard diagnostic plots and statistical indicators. Independence of errors was assessed with the Durbin–Watson statistic, with values close to 2 indicating no violation. Multicollinearity was assessed by Tolerance and Variance Inflation Factor (see Supplementary Tables S3, S4), with all values within acceptable thresholds (Tolerance > 0.20; VIF < 5.0). Linearity, normality, and homoscedasticity were supported by diagnostic checks, and representative residual-vs.-fitted and P–P plots shown in Supplementary Figures 1–4.

All statistical analyses were performed using IBM SPSS Statistics (Version 29). A significance level of α = 0.05 (two-tailed) was applied for all inferential tests. Exact *p*-values are reported unless *p* < 0.001. Partial eta-squared (η^2^) was reported as a measure of effect size for MANOVA and univariate follow-up tests. For regression analyses, standardized beta coefficients and the coefficient of determination (*R*^2^) were reported. Effect sizes were interpreted following Cohen's (1988) guidelines: η^2^ (0.01 = small, 0.06 = medium, 0.14 = large), β (0.10 = small, 0.30 = medium, 0.50 = large), and *R*^2^ (0.02 = small, 0.13 = medium, 0.26 = large).

## Results

The first MANOVA revealed statistically significant multivariate group differences in perceived stress depending on mental health status, Wilks' Λ = 0.84, *F*_(7,159)_ = 4.26, *p* < 0.001, with a large effect size (partial η^2^ = 0.16), indicating that the groups differ across the combination of stress domains. Follow-up univariate analyses showed that adolescents with mental health problems reported significantly higher stress perception across all life domains than those without mental health problems, with effect sizes ranging from small to large (partial η^2^ = 0.04 to 0.14; Table 1).

**Table 1:** Group differences in perceived stress across seven life domains by mental health status.

**Stress domain**	**Mental health status**	***M***	***SD***	***n***	***F*_(1,165)_**	***p***	**partial η^2^**
School	Clinical	2.61	0.88	84	9.38	0.003	0.05
	Healthy controls	2.25	0.65	87			
Future	Clinical	2.96	0.77	84	6.89	0.009	0.04
	Healthy controls	2.64	0.77	87			
Parents	Clinical	2.01	0.87	84	6.20	0.014	0.04
	Healthy controls	1.72	0.65	87			
Peers	Clinical	2.50	0.83	84	9.85	0.002	0.06
	Healthy controls	2.14	0.65	87			
Leisure	Clinical	2.35	0.76	84	14.81	<0.001	0.08
	Healthy controls	1.95	0.56	87			
Romantic Relationships	Clinical	2.27	0.85	84	9.47	0.002	0.05
	Healthy controls	1.91	0.65	87			
Self-related Stress	Clinical	2.56	0.79	84	27.04	<0.001	0.14
	Healthy controls	2.01	0.57	87			

The comparison was based on mental health status as classified by the PHQ-D (Löwe et al., 2002). The mentally healthy control group included only adolescents without indications of a current psychological syndrome, while the clinical group comprised those who met the criteria for at least one psychological syndrome according to PHQ-D (Löwe et al., 2002).

The second MANOVA showed statistically significant multivariate group differences in coping styles depending on mental health status, Wilks' Λ =0.80, *F*_(3,164)_ = 13.95, *p* < 0.001, with a large effect size (partial η^2^ = 0.20), indicating that the groups also differed in their overall coping patterns. Follow-up univariate analyses showed significant group differences in all three coping styles, with effect sizes ranging from small to large (partial η^2^ = 0.03 to 0.14; Table 2).

**Table 2:** Group differences in coping style by mental health status.

**Coping Style**	**Mental health status**	***M***	***SD***	***n***	***F*_(1,166)_**	***p***	**partial η^2^**
Active	Clinical	19.65	10.12	84	4.31	0.039	0.03
	Healthy controls	22.80	9.59	87			
Internal	Clinical	19.55	8.75	84	4.61	0.033	0.03
	Healthy controls	22.56	9.39	87			
Withdrawal	Clinical	17.11	10.72	84	27.46	<0.001	0.14
	Healthy controls	9.69	7.41	87			

The comparison was based on mental health status as classified by the PHQ-D (Löwe et al., 2002). The mentally healthy control group included only adolescents without indications of a current psychological syndrome, while the clinical group comprised those who met the criteria for at least one psychological syndrome according to PHQ-D (Löwe et al., 2002).

To examine group-specific associations between psychodynamic characteristics and adolescents' stress perception and coping behavior, separate stepwise linear regression analyses were conducted for adolescents with and without mental health problems. The full results are presented in Table 3 (clinical sample) and Table 4 (non-clinical sample).

**Table 3:** Stepwise linear regression analyses predicting stress and dysfunctional coping style in adolescents with mental health problems.

**Outcome variable**	**Predictor**	***B***	***SE***	**β**	***t***	***p***	**95% *CI* for *B***	***R^2^***	***F***	***p* (model)**	***DW***
School-related stress	Structural identity impairment	0.40	0.15	0.31	2.67	0.010^*^	(0.10; 0.71)	0.40	14.01	<0.001	1.90
	Guilt conflict in passive mode	0.26	0.08	0.31	3.08	0.003^**^	(0.09; 0.42)				
	Guilt conflict in active mode	0.41	0.17	0.27	2.40	0.020^*^	(0.07; 0.76)				
Future-related stress	Structural identity impairment	0.23	0.07	0.35	3.33	<0.001^***^	(0.09; 0.36)	0.38	13.10	<0.001	2.13
	Self-worth conflict in passive mode	0.20	0.08	0.27	2.55	0.013^*^	(0.04; 0.35)				
	Guilt conflict in passive mode	0.17	0.07	0.25	2.49	0.015^*^	(0.03; 0.31)				
Family-related stress	Guilt conflict in active mode	0.58	0.17	0.36	3.39	0.001^**^	(0.24; 0.92)	0.39	13.42	<0.001	1.73
	Closeness vs. distance conflict in active mode	0.49	0.19	0.26	2.53	0.014^*^	(0.10; 0.87)				
	Taking care of oneself vs. being cared for conflict in active mode	0.30	0.12	0.25	2.46	0.017^*^	(0.06; 0.55)				
Peer relationship stress	Structural identity impairment	0.98	0.13	0.82	7.60	<0.001^***^	(0.72; 1.23)	0.53	29.31	<0.001	2.12
	Oedipal conflict in active mode	0.25	0.07	0.32	3.72	<0.001^***^	(0.12; 0.38)				
	Maladaptive defense style	−0.25	0.09	−0.30	−2.73	0.008^**^	(−0.44; −0.07)				
Leisure-related stress	Structural identity impairment	0.53	0.11	0.49	4.60	<0.001^***^	(0.30; 0.75)	0.24	21.19	<0.001	1.89
Romantic relationship stress	Structural identity impairment	0.51	0.13	0.41	3.82	<0.001^***^	(0.24; 0.77)	0.26	11.61	<0.001	2.22
	Oedipal conflict in passive mode	0.23	0.10	0.25	2.32	0.023^*^	(0.03; 0.44)				
Self-related stress	Total structural impairment	1.15	0.12	0.94	9.33	<0.001^***^	(0.90; 1.39)	0.61	51.37	<0.001	2.19
	Maladaptive defense style	−0.23	0.08	−0.29	−2.89	0.005^**^	(−0.39; −0.07)				
Dysfunctional coping (withdrawal)	Structural attachment impairment	5.78	1.72	0.38	3.36	0.001^**^	(2.35; 9.21)	0.24	10.45	<0.001	1.50
	Closeness vs. distance conflict in active mode	5.09	2.37	0.24	2.15	0.036^*^	(0.35; 9.82)				

*N* = 84. *B*, unstandardized regression coefficient; *SE*, standard error of B; β, standardized regression coefficient; *CI*, confidence interval; *R*^2^, coefficient of determination; *F, F*-statistic for overall model significance; *DW*, Durbin–Watson statistic (residual autocorrelation).

To account for possible confounding influences, age, gender, and SES were included as additional predictors in all models. All variables were entered using stepwise regression; only predictors retained in the final model are displayed. Corresponding tolerance and variance inflation factor (VIF) values for all predictors were within commonly accepted thresholds (Tolerance > 0.20; VIF < 5.0) and are provided in Supplementary Table S3.

Significant predictors are indicated by asterisks (

^*^p < 0.05;

^**^p < 0.01;

^***^p < 0.001).

**Table 4:** Stepwise linear regression analyses predicting stress and functional coping style in adolescents without mental health problems.

**Outcome Variable**	**Predictor**	***B***	***SE***	**β**	***t***	***p***	**95% *CI* for *B***	***R^2^***	***F***	***p* (model)**	***DW***
School-related stress	Age	0.08	0.03	0.29	2.56	0.013^*^	(0.02; 0.14)	0.08	6.55	0.013	2.23
Future-related stress	Structural identity impairment	0.73	0.14	0.53	5.38	<0.001^***^	(0.46; 0.99)	0.28	28.91	<0.001	2.35
Family-related stress	Structural attachment impairment	0.50	0.15	0.37	3.43	0.001^**^	(0.21; 0.80)	0.14	11.73	0.001	2.16
Peer relationship stress	Structural identity impairment	0.71	0.11	0.61	6.62	<0.001^***^	(0.49; 0.92)	0.37	43.77	<0.001	2.41
Leisure-related stress	Maladaptive defense style	0.19	0.06	0.36	3.17	0.002^**^	(0.07; 0.32)	0.40	24.10	<0.001	2.11
	Structural identity impairment	0.35	0.11	0.34	3.02	0.004^**^	(0.12; 0.57)				
Romantic relationship stress	Structural identity impairment	0.57	0.13	0.45	4.51	<0.001^***^	(0.32; 0.82)	0.29	14.99	<0.001	1.50
	Age	0.06	0.03	0.22	2.19	0.032^*^	(0.01; 0.12)				
Self-related stress	Structural identity impairment	0.81	0.07	0.76	11.33	<0.001^***^	(0.67; 0.95)	0.70	56.30	<0.001	2.05
	Age	0.04	0.02	0.15	2.26	0.027^*^	(0.01; 0.07)				
	Sex	−0.17	0.07	−0.15	−2.25	0.028^*^	(−0.31; −0.02)				
Functional coping (active coping strategies)	Sex	−6.68	2.14	−0.34	−3.12	0.003^**^	(−10.94; −2.41)	0.16	6.82	0.002	1.75
	Structural attachment impairment	−5.45	2.32	−0.25	−2.34	0.022^*^	(−10.08; −0.82)				

*N* = 87. *B*, unstandardized regression coefficient; *SE*, standard error of B; β, standardized regression coefficient; *CI*, confidence interval; *R*^2^, coefficient of determination; *F, F*-statistic for overall model significance; *DW*, Durbin–Watson statistic (residual autocorrelation).

To account for possible confounding influences, age, gender, and SES were included as additional predictors in all models.

All variables were entered using stepwise regression; only predictors retained in the final model are displayed. Corresponding tolerance and variance inflation factor (VIF) values for all predictors were within commonly accepted thresholds (Tolerance > 0.20; VIF < 5.0) and are provided in Supplementary Table S4.

Significant predictors are indicated by asterisks (

^*^p < 0.05;

^**^p < 0.01;

^***^p < 0.001).

Among adolescents with mental health problems, higher stress levels across various life domains were consistently predicted by impairments in personality structure—especially identity-related impairments—as well as by specific psychodynamic conflicts (e.g., guilt and closeness vs. distance conflict). The highest explained variance was found for self-related stress (*R*^2^ = 0.61), which was primarily predicted by total structural impairment and maladaptive defense style. Notably, across several models, maladaptive defense style was negatively associated with perceived stress. Dysfunctional coping (withdrawal) was predicted by structural impairments in attachment and active closeness vs. distance conflict.

In contrast, among adolescents without mental health problems, psychodynamic variables showed a weaker but still meaningful association with perceived stress. Structural identity impairments emerged as consistent predictors of stress, particularly in self- and peer-related domains. Active coping was negatively associated with structural impairments and adolescents' sex, indicating that female adolescents reported higher levels of active coping. No significant predictors were identified for internal coping in the non-clinical group.

Additional analyses excluding the neurotic defense style (ω = 0.50) are reported in Supplementary Tables S5, S6. The overall pattern of significant predictors remained unchanged, supporting the robustness of the regression results.

## Discussion

This study aimed to examine whether psychodynamic factors are associated with differences in how adolescents—with and without mental health problems—perceive and manage stress. Our findings highlight the relevance of psychodynamic characteristics—particularly impairments in personality structure, psychodynamic conflicts, and defense styles—in relation to adolescents' subjective stress experiences and coping behaviors.

First of all, consistent with prior findings in adolescent populations, higher perceived stress across various life domains has been repeatedly observed among adolescents experiencing mental health problems compared to their mentally healthy peers (Seiffge-Krenke et al., 2009; Meng et al., 2011; Su et al., 2022). Our results thus corroborate this well-established pattern, reinforcing the link between elevated stress perception and psychopathology during adolescence.

Regarding coping styles, our findings revealed significant differences between the two groups. Adolescents with mental health problems reported lower levels of functional (active and internal) coping and higher levels of withdrawal, reflecting the tendency toward dysfunctional, avoidant coping observed in clinical populations (Beyer and Lohaus, 2007; Huemer et al., 2015). In contrast, adolescents without mental health problems demonstrated more frequent use of functional coping strategies, which are linked to better stress regulation and psychological adjustment (Seiffge-Krenke, 1995; Gelhaar et al., 2007). These findings highlight that mental health status is strongly associated with coping behavior during adolescence and underscore the importance of promoting functional coping mechanisms in vulnerable youth.

Building on the observed group differences in stress and coping, the subsequent regression analyses examined the associations between psychodynamic factors and adolescents' stress perception and coping behavior. Among adolescents with mental health problems, the results suggest that impairments in personality structure—particularly in the identity domain—and psychodynamic conflicts are prominently related to heightened stress perception across multiple life domains. This supports core assumptions of psychodynamic theory, which posits that early-formed vulnerabilities in personality structure and unresolved inner conflicts can limit an individual's capacity to process and regulate stress, thereby increasing the likelihood of experiencing heightened stress (Task Force OPD-CA-2 (Ed.), 2020).

Impairments in the personality structure domain of identity emerged as the most consistent predictor and were significantly associated with elevated stress perception in nearly all domains. In the OPD-CA framework (Task Force OPD-CA-2 (Ed.),2020), identity refers to the capacity to perceive oneself and others as differentiated and temporally stable entities. When this capacity is impaired, adolescents may struggle to integrate emotional experiences and interpersonal demands, thereby increasing their vulnerability to stress. This finding aligns with prior research showing that adolescents characterized by diffuse or unstable identity statuses—such as identity diffusion—report higher levels of psychological distress and stress compared to those with more consolidated identity development (Verschueren et al., 2017). However, due to the cross-sectional design, it remains unclear whether impaired identity leads to increased stress or vice versa. It is equally plausible that heightened stress experiences—particularly in developmental contexts that challenge self-definition—may erode identity consolidation over time. Thus, reciprocal models in which impaired identity and stress mutually reinforce each other appear conceivable and should be addressed in longitudinal studies.

In addition, psychodynamic conflicts exhibited domain-specific associations with stress perception among adolescents with mental health problems. For example, the guilt conflict was linked to increased stress perception related to school, future, and family. The guilt conflict involves a troubled ability to assign blame realistically, leading to excessive self-reproach in passive mode or denial of guilt in active mode (Task Force OPD-CA-2 (Ed.), 2020). In the context of school and future-related demands, adolescents with a passive guilt conflict may internalize failure or uncertainty as personal shortcomings, resulting in heightened stress. Similarly, existing relationship tensions or conflicts within the family could lead to increased accusations of guilt and the feeling of being treated unfairly, which in turn can increase the experience of stress in the family context—especially in adolescence, when autonomy and loyalty to the family are restructured.

Another example underscoring the relevance of psychodynamic conflicts for stress perception among adolescents with mental health problems is the passive oedipal conflict, which was associated with increased stress in romantic relationships. In the context of the passive mode, this conflict involves a repressed experience of one's own sexual identity and erotic desires, leading to insecurity, and avoidance in intimate relational contexts (Task Force OPD-CA-2 (Ed.), 2020). During adolescence—a developmental stage marked by the exploration of romantic and sexual identity (Blos, 1962)—such unresolved inner conflict may lead to heightened emotional tension and self-doubt in close relationships, thereby contributing to increased stress in contexts involving romantic intimacy.

Furthermore, the active closeness vs. distance conflict was associated with family-related stress. In this mode, adolescents strive for emotional independence and autonomy, often by distancing themselves from attachment figures (Task Force OPD-CA-2 (Ed.), 2020). Empirical studies have shown that increased parent–child conflict during adolescence is associated with reduced emotional closeness and heightened relational stress (Branje et al., 2012). This aligns with psychodynamic theory, which posits that early internalized conflicts surrounding closeness and autonomy may be reactivated in family contexts during adolescence (Nicolò, 2018), thereby amplifying stress when attachment and independence must be renegotiated.

Due to the cross-sectional design of the study, it cannot be ruled out that the observed associations also operate in the opposite direction. That is, elevated stress experiences in various life domains may, in turn, reinforce the significance and intensity of psychodynamic conflicts. For instance, school- or family-related stress could exacerbate the guilt conflict by triggering self-blame or perceived unfairness, while relational burdens in romantic contexts might activate the passive oedipal conflict through heightened sexual insecurities. This points to a bidirectional dynamic in which unresolved conflicts not only amplify stress reactivity but perceived stress also activates or intensifies psychodynamic conflicts—a circular process that may escalate over time. Prospective studies are needed to disentangle these dynamics and clarify their temporal sequencing.

Contrary to our theoretical expectations, a negative association emerged in the clinical sample between maladaptive defense style and the level of peer-related and self-related stress. This finding suggests that adolescents with more pronounced immature defense mechanisms tend to report lower subjective stress in these domains. One explanation can be found in Gil's (2005) study, which shows that mentally stressed children and adolescents may develop a repressive defense style—cognitively and emotionally splitting off stressors without consciously perceiving them. Although objective stressors (e.g., social insecurity or negative self-image) remain present, this defense style leads to a distorted self-perception, whereby internal stress may be underreported or even not consciously experienced. In this sense, immature defense style may not only reflect psychological burden but also distort its conscious awareness, as seen in maladaptive defense mechanisms such as affect isolation, splitting, or dissociation.

Beyond this psychodynamic interpretation, measurement-related factors must also be considered. The assessment of defense mechanisms via self-report is inherently challenging, given that these processes are largely unconscious and may elude introspection. Moreover, response biases such as social desirability or underreporting can further distort self-ratings, potentially explaining inverse associations between maladaptive defense styles and perceived stress. To address these limitations, future research should combine self-report with qualitative approaches and multi-informant perspectives (e.g., parents, teachers, and clinicians), which could help to clarify whether such findings reflect genuine psychological processes or methodological artifacts.

Notably, in the clinical sample, dysfunctional coping behavior was positively associated with impairments in the personality structure domain of attachment as well as with the active mode of the closeness versus distance conflict. These associations point to the potential relevance of early relational experiences and internalized attachment patterns for adolescents' coping behavior. Specifically, impairments in attachment abilities—such as difficulties in trusting others, regulating emotions in relationships, or maintaining stable bonds (Task Force OPD-CA-2 (Ed.), 2020)—may limit adolescents' ability to seek and make use of social support, possibly increasing the use of withdrawal or avoidance strategies. Furthermore, the active mode of the closeness vs. distance conflict—marked by a struggle for emotional autonomy through distancing from attachment figures (Task Force OPD-CA-2 (Ed.), 2020)–was not only associated with higher family-related stress (see above), but also with dysfunctional behaviors such as withdrawal. Taken together, these findings align with psychodynamic perspectives that emphasize the central importance of early attachment dynamics for the development of self-regulation and coping (Fonagy and Target, 2002), and suggest that attachment-related vulnerabilities may be relevant targets in interventions aimed at supporting adolescents with mental health problems. Again, it should be noted that due to the cross-sectional design of the study, causal directions cannot be established. Thus, it remains unclear whether impairments in attachment and the active closeness vs. distance conflict contribute to dysfunctional coping behaviors, or whether such coping patterns in turn exacerbate attachment difficulties and relational tensions.

In the group of mentally healthy adolescents, impairments in the personality structure domains of identity and attachment emerged as significant predictors of stress perception across various life domains. Specifically, identity impairments significantly predicted stress related to the future, peers, romantic relationships, and the self. Attachment was particularly relevant for family-related stress and also served as a negative predictor of functional coping, meaning that greater attachment impairments were associated with less active, functional coping strategies. A similar association was found in the clinical group, where attachment impairments, together with the active closeness vs. distance conflict, positively predicted dysfunctional coping (see above). These parallel findings highlight the central importance of attachment abilities for stress regulation during adolescence and suggest that prevention or therapeutic intervention targeting negative early attachment experiences could substantially contribute both to the development of functional and to the reduction of dysfunctional coping mechanisms. To build on these results, further research is needed, including developmental longitudinal studies as well as specialized attachment-focused intervention trials.

Unexpectedly, no significant psychodynamic predictors emerged for internal coping. This absence of associations may suggest that internal strategies such as cognitive problem solving are less influenced by deeper personality structure or conflict patterns in psychologically resilient adolescents. However, it may also reflect limitations in current measurement approaches or sample-specific effects. Further research is needed to better understand the developmental and contextual factors that shape internal coping mechanisms in adolescence, particularly in relation to intrapsychic functioning.

Further, among the mentally healthy adolescents, age was also found to influence specific stress domains such as school-related, romantic, and self-related stress, which aligns with the developmental challenges of adolescence (Compas et al., 2001; Lazarus and Folkman, 1984). Gender differences appeared in self-related stress and functional coping, with female adolescents tending to show more active coping but also reporting somewhat higher self-related stress. These findings are consistent with previous research indicating that adolescent girls often experience higher internalizing symptoms and stress levels (Nolen-Hoeksema and Girgus, 1994) and tend to engage more frequently in social support seeking and other active coping behaviors compared to boys (Tamres et al., 2002).

Overall, this study underscores the significant associations between psychodynamic factors—personality structure, psychodynamic conflicts, and defense styles—and adolescents' perception of stress and their coping behaviors. Our findings extend existing research by demonstrating that these intrapsychic characteristics are relevant not only among adolescents with mental health problems but also in their mentally healthy peers, highlighting the ubiquitous relevance of underlying psychodynamic processes during this critical developmental stage. Particularly, impairments in identity and attachment domains of personality structure consistently predicted stress perception and coping styles across both groups, emphasizing the centrality of these constructs for adaptive psychological functioning in adolescence.

Building on these findings, personality structure and attachment-focused prevention and intervention strategies appear especially promising. Mentalization-Based Treatment for Adolescents (MBT-A), for instance, has been shown to strengthen personality structure capacities such as affect regulation, self-other differentiation, and interpersonal understanding (Byrne et al., 2020). Moreover, adolescents participating in MBT-A programs exhibit higher rates of secure attachment compared with control groups (Kobak et al., 2015), pointing to the high potential of such approaches in addressing stress- and coping-related vulnerabilities identified in our study. Additionally, school- or community-based psychodynamic counseling programs may provide accessible contexts for fostering personality structure abilities, enhancing attachment security, and promoting functional coping strategies. Implementing such interventions in educational or youth-centered settings could help mitigate the effects of impaired attachment and psychodynamic conflicts on stress perception and coping in vulnerable adolescents.

Nonetheless, several limitations must be acknowledged. First, the cross-sectional design limits causal interpretations and precludes conclusions about developmental trajectories or directionality between psychodynamic factors, stress perception, and coping behaviors. Future longitudinal research could explicitly model reciprocal pathways, for example by testing whether psychodynamic vulnerabilities predict subsequent stress reactivity and coping impairments, or whether chronic stress exposure in turn exacerbates structural impairments and psychodynamic conflicts. Second, the reliance on self-report measures may introduce response biases such as social desirability or limited insight, particularly for complex constructs like psychodynamic conflicts and defense mechanisms. Moreover, the reported reliabilities for psychodynamic conflicts were very high (Spearman–Brown >0.99), suggesting possible redundancy due to strong inter-item correlations, which may lead to an overestimation of internal consistency and should be considered when interpreting associations. Conversely, the neurotic defense style scale showed relatively low internal consistency (ω = 0.50), which may have limited the reliability of regression estimates and could partly explain the absence of significant associations for this construct, indicating cautious interpretation. Third, the clinical group was defined based on self-reported symptomatology rather than structured clinical interviews, affecting the specificity and generalizability of findings to clinical populations. The sample size, while adequate for the analyses performed, may limit statistical power for detecting smaller effects and subgroup analyses. Furthermore, the high proportion of adolescents from higher socioeconomic backgrounds may restrict generalizability to lower SES populations, who may face different stressors and coping demands. Although both clinical and control participants were recruited through the same sources (schools and youth community centers), differences in social background or stress exposure cannot be fully ruled out. Finally, some regression models—particularly those predicting self-related stress—showed relatively high R^2^ values. While all regression assumptions were met, this may reflect the conceptual interrelatedness of OPD-CA domains, the relative homogeneity of the clinical and non-clinical samples regarding stress and psychodynamic characteristics, or the exploratory nature of the stepwise regression approach.

Future research would benefit from longitudinal designs to clarify developmental pathways and potential sensitive periods for intervention. Intervention studies targeting attachment-related impairments and specific psychodynamic conflicts are also warranted to assess efficacy in enhancing active and reducing avoidant coping. Incorporating multi-informant assessments and structured clinical interviews would strengthen validity, and expanding samples to diverse cultural and socioeconomic backgrounds could improve generalizability and inform culturally sensitive approaches.

## Data Availability

The raw data supporting the conclusions of this article will be made available by the authors, without undue reservation. Requests to access the datasets should be directed to the corresponding author.
