# Supplementary material for: Understanding adolescent stress and coping through psychodynamic constructs: evidence from a comparative study
Source: Front Psychol. 2025 Sep 23;16:1668051. doi: 10.3389/fpsyg.2025.1668051 (PMC12503032; doi:10.3389/fpsyg.2025.1668051)
Supplement: Supplementary file 1 [file Supplementary_file_1.docx]

Supplementary Material

# Supplementary Tables and Figures

## Supplementary Tables

**Table S1.** *Recruitment Sources by Group*

| **Recruitment Source** | **Clinical Group (*n* = 84)** | **Non-Clinical Group (*n* = 87)** | **Total** |
| --- | --- | --- | --- |
| Schools | 24 (28.6%) | 27 (31.0%) | 51 |
| Youth Centers | 60 (71.4%) | 60 (69.0%) | 120 |
| **Total** | 84 | 87 | 171 |

***Note****.* This table presents the distribution of participants across recruitment sources by group. Values are shown as absolute numbers (*n*); percentages are provided for additional clarity. Recruitment occurred within the same time frame to minimize potential bias. All participants were recruited in Germany.

**Table S2.** *Theoretically Guided Regression Models Predicting Stress & Dysfunctional Coping in Adolescents With Mental Health Problems*

| **Outcome Variable** | **Predictor** | ***B*** | ***SE*** | **β** | ***t*** | ***p*** | **95% *CI* for *B*** | **Tolerance** | **VIF** | ***R²*** | ***F*** | ***p* (model)** | ***DW*** |
| --- | --- | --- | --- | --- | --- | --- | --- | --- | --- | --- | --- | --- | --- |
| Family-related stress | **Closeness vs. distance conflict in active mode** | **0.50** | **0.20** | **0.27** | **2.46** | **.017*** | **[0.10; 0.91]** | **0.83** | **1.20** | 0.43 | 5.48 | <.001 | 2.21 |
|  | Closeness vs. distance conflict in passive mode | -0.08 | 0.09 | -0.10 | -0.90 | .372 | [-0.27; 0.10] | 0.81 | 1.24 |  |  |  |  |
|  | **Taking care of oneself vs. being cared for conflict in active mode** | **0.27** | **0.13** | **0.23** | **2.12** | **.038*** | **[0.02; 0.53]** | **0.88** | **1.14** |  |  |  |  |
|  | Taking care of oneself vs. being cared for conflict in passive mode | -0.16 | 0.12 | -0.14 | -1.30 | .202 | [-0.40; 0.09] | 0.85 | 1.17 |  |  |  |  |
|  | **Guilt conflict in active mode** | **0.64** | **0.18** | **0.40** | **3.65** | **<.001***** | **[0.29; 0.99]** | **0.82** | **1.23** |  |  |  |  |
|  | Guilt conflict in passive mode | 0.11 | 0.09 | 0.13 | 1.22 | .227 | [-0.07; 0.29] | 0.87 | 1.14 |  |  |  |  |
|  | Age | -0.01 | 0.04 | -0.02 | -0.23 | .820 | [-0.10; 0.08] | 0.92 | 1.08 |  |  |  |  |
|  | SES | -0.01 | 0.01 | -0.04 | -0.37 | .711 | [-0.01; 0.01] | 0.90 | 1.11 |  |  |  |  |
| Romantic relationship stress | Oedipal conflict in active mode | 0.01 | 0.09 | 0.01 | 0.10 | .924 | [-0.17; 0.19] | 1.00 | 1.00 | 0.06 | 2.51 | .088 | 1.87 |
|  | **Oedipal conflict in passive mode** | **0.22** | **0.10** | **0.24** | **2.24** | **.028*** | **[0.03; 0.42]** | **1.00** | **1.00** |  |  |  |  |
| Self-related stress | **Total structural impairment** | **1.17** | **0.12** | **0.97** | **1.00** | **<.001***** | **[0.94; 1.40]** | **0.65** | **1.54** | 0.67 | 53.63 | <.001 | 2.22 |
|  | **Maladaptive defense style** | **-0.21** | **0.07** | **-0.26** | **-3.15** | **.002**** | **[-0.34; -0.07]** | **0.62** | **1.61** |  |  |  |  |
|  | Self-worth conflict in passive mode | -0.02 | 0.06 | -0.03 | -0.36 | .720 | [-0.13; 0.09] | 0.66 | 1.52 |  |  |  |  |
| Dys-functional coping (withdrawal) | Maladaptive defense style | 1.92 | 1.39 | 0.17 | 1.38 | .171 | [-0.85; 4.70] | 0.60 | 1.67 | 0.30 | 4.56 | <.001 | 2.39 |
|  | **Structural attachment impairment** | **6.00** | **3.00** | **0.39** | **2.00** | **.049*** | **[0.03; 11.98]** | **0.65** | **1.54** |  |  |  |  |
|  | Structural identity impairment | -0.20 | 2.90 | -0.01 | -0.07 | .945 | [-5.98; 5.57] | 0.65 | 1.54 |  |  |  |  |
|  | Structural interpersonality impairment | -1.22 | 3.69 | -0.08 | -0.33 | .742 | [-8.58; 6.14] | 0.50 | 2.00 |  |  |  |  |
|  | Structural control impairment | -1.77 | 2.65 | -0.13 | -0.67 | .508 | [-7.05; 3.52] | 0.57 | 1.75 |  |  |  |  |
|  | **Closeness vs. distance conflict in active mode** | **5.29** | **2.06** | **0.29** | **2.57** | **.012*** | **[1.18; 9.40]** | **0.75** | **1.33** |  |  |  |  |
|  | Closeness vs. distance conflict in passive mode | 1.15 | 1.09 | 0.12 | 1.05 | .297 | [-1.03; 3.33] | 0.73 | 1.37 |  |  |  |  |

***Note*.** *N* = 84. *B* = unstandardized regression coefficient; *SE* = standard error of B; β = standardized regression coefficient; *CI* = confidence interval; VIF = Variance Inflation Factor**;** *R²* = coefficient of determination; *F* = *F*-statistic for overall model significance; *DW* = Durbin–Watson statistic (residual autocorrelation). Predictors were selected in line with psychodynamic theory and informed by clinical considerations, reflecting conceptually guided hypotheses. All predictors were entered simultaneously using forced entry. Tolerance and VIF values were examined to assess multicollinearity. Following common criteria (Tolerance > 0.20; VIF < 5.0), all reported models met these thresholds, indicating that multicollinearity was not a concern.

Significant predictors are highlighted in bold and indicated by asterisks (**p* < .05; ***p* < .01; ****p* < .001).

**Table S3.** *Multicollinearity Diagnostics: Clinical Sample*

| **Outcome Variable** | **Predictor** | **Tolerance** | **VIF** |
| --- | --- | --- | --- |
| School-related stress | Structural identity impairment | 0.70 | 1.43 |
|  | Guilt conflict in passive mode | 0.92 | 1.09 |
|  | Guilt conflict in active mode | 0.75 | 1.33 |
| Future-related stress | Structural identity impairment | 0.87 | 1.15 |
|  | Self-worth conflict in passive mode | 0.84 | 1.19 |
|  | Guilt conflict in passive mode | 0.95 | 1.05 |
| Family-related stress | Guilt conflict in active mode | 0.86 | 1.17 |
|  | Closeness vs. distance conflict in active mode | 0.92 | 1.08 |
|  | Taking care of oneself vs. being cared for conflict in active mode | 0.90 | 1.12 |
| Peer relationship stress | Structural identity impairment | 0.63 | 1.59 |
|  | Oedipal conflict in active mode | 0.98 | 1.03 |
|  | Maladaptive defense style | 0.63 | 1.59 |
| Leisure-related stress | Structural identity impairment | 1.00 | 1.00 |
| Romantic relationship stress | Structural identity impairment | 0.98 | 1.02 |
|  | Oedipal conflict in passive mode | 0.98 | 1.02 |
| Self-related stress | Total structural impairment | 0.70 | 1.43 |
|  | Maladaptive defense style | 0.70 | 1.43 |
| Dysfunctional coping (withdrawal) | Structural attachment impairment | 0.93 | 1.07 |
|  | Closeness vs. distance conflict in active mode | 0.93 | 1.07 |

***Note*.** *N* = 84. VIF = Variance Inflation Factor. Tolerance and VIF values were inspected for each predictor. All values are within commonly accepted thresholds (Tolerance > 0.20; VIF < 5.0), indicating no multicollinearity concerns.

| **Outcome Variable** | **Predictor** | **Tolerance** | **VIF** |
| --- | --- | --- | --- |
| School-related stress | Age | 1.00 | 1.00 |
| Future-related stress | Structural identity impairment | 1.00 | 1.00 |
| Family-related stress | Structural attachment impairment | 1.00 | 1.00 |
| Peer relationship stress | Structural identity impairment | 1.00 | 1.00 |
| Leisure-related stress | Maladaptive defense style | 0.64 | 1.57 |
|  | Structural identity impairment | 0.64 | 1.57 |
| Romantic relationship stress | Structural identity impairment | 0.96 | 1.04 |
|  | Age | 0.96 | 1.04 |
| Self-related stress | Structural identity impairment | 0.93 | 1.07 |
|  | Age | 0.96 | 1.04 |
|  | Sex | 0.97 | 1.04 |
| Functional coping (active coping strategies) | Sex | 0.99 | 1.02 |
|  | Structural attachment impairment | 0.99 | 1.02 |

**Table S4.** *Multicollinearity Diagnostics: Non-Clinical Sample*

***Note*.** *N* = 87. VIF = Variance Inflation Factor. Tolerance and VIF values were inspected for each predictor. All values are within commonly accepted thresholds (Tolerance > 0.20; VIF < 5.0), indicating no multicollinearity concerns.

| **Outcome Variable** | **Predictor** | **β (full model)** | ***p* (full model)** | **β (without neurotic defense style)** | ***p* (without neurotic defense style)** |
| --- | --- | --- | --- | --- | --- |
| School-related stress | Structural identity impairment | 0.31 | .010* | 0.31 | .010* |
|  | Guilt conflict in passive mode | 0.31 | .003** | 0.31 | .003** |
|  | Guilt conflict in active mode | 0.27 | .020* | 0.27 | .020* |
| Future-related stress | Structural identity impairment | 0.35 | <.001*** | 0.35 | <.001*** |
|  | Self-worth conflict in passive mode | 0.27 | .013* | 0.27 | .013* |
|  | Guilt conflict in passive mode | 0.25 | .015* | 0.25 | .015* |
| Family-related stress | Guilt conflict in active mode | 0.36 | .001** | 0.36 | .001** |
|  | Closeness vs. distance conflict in active mode | 0.26 | .014* | 0.26 | .014* |
|  | Taking care of oneself vs. being cared for conflict in active mode | 0.25 | .017* | 0.25 | .017* |
| Peer relationship stress | Structural identity impairment | 0.82 | <.001*** | 0.82 | <.001*** |
|  | Oedipal conflict in active mode | 0.32 | <.001*** | 0.32 | <.001*** |
|  | Maladaptive defense style | -0.30 | .008** | -0.30 | .008** |
| Leisure-related stress | Structural identity impairment | 0.49 | <.001*** | 0.49 | <.001*** |
| Romantic relationship stress | Structural identity impairment | 0.41 | <.001*** | 0.41 | <.001*** |
|  | Oedipal conflict in passive mode | 0.25 | .023* | 0.25 | .023* |
| Self-related stress | Total structural impairment | 0.94 | <.001*** | 0.94 | <.001*** |
|  | Maladaptive defense style | -0.29 | .005** | -0.29 | .005** |
| Dysfunctional coping (withdrawal) | Structural attachment impairment | 0.38 | .001** | 0.38 | .001** |
|  | Closeness vs. distance conflict in active mode | 0.24 | .036* | 0.24 | .036* |

**Table S5.** *Robustness Check: Excluding Neurotic Defense Style in the Models Predicting Stress & Coping in the Clinical Sample*

***Note*.** *N* = 84. Values are standardized regression coefficients (β) with corresponding p-values. The table compares models including versus excluding the neurotic defense style, showing that the results remain unchanged. Age, gender, and SES were included as additional predictors in all models. All variables were entered using stepwise regression; only predictors retained in the final model are displayed.

Significant predictors are indicated by asterisks (**p* < .05; ***p* < .01; ****p* < .001).

**Table S6.** *Robustness Check: Excluding Neurotic Defense Style in the Models Predicting Stress and Coping in the Non-Clinical Sample*

| **Outcome Variable** | **Predictor** | **β (full model)** | ***p* (full model)** | **β (without neurotic defense style)** | ***p* (without neurotic defense style)** |
| --- | --- | --- | --- | --- | --- |
| School-related stress | Age | 0.29 | .013* | 0.29 | .013* |
| Future-related stress | Structural identity impairment | 0.53 | <.001*** | 0.53 | <.001*** |
| Family-related stress | Structural attachment impairment | 0.37 | .001** | 0.37 | .001** |
| Peer relationship stress | Structural identity impairment | 0.61 | <.001*** | 0.61 | <.001*** |
| Leisure-related stress | Maladaptive defense style | 0.36 | .002** | 0.36 | .002** |
|  | Structural identity impairment | 0.34 | .004** | 0.34 | .004** |
| Romantic relationship stress | Structural identity impairment | 0.45 | <.001*** | 0.45 | <.001*** |
|  | Age | 0.22 | .032* | 0.22 | .032* |
| Self-related stress | Structural identity impairment | 0.76 | <.001*** | 0.76 | <.001*** |
|  | Age | 0.15 | .027* | 0.15 | .027* |
|  | Sex | -0.15 | .028* | -0.15 | .028* |
| Functional coping (active coping strategies) | Sex | -0.34 | .003** | -0.34 | .003** |
|  | Structural attachment impairment | -0.25 | .022* | -0.25 | .022* |

***Note*.** *N* = 87. Values are standardized regression coefficients (β) with corresponding p-values. The table compares models including versus excluding the neurotic defense style, showing that the results remain unchanged. Age, gender, and SES were included as additional predictors in all models. All variables were entered using stepwise regression; only predictors retained in the final model are displayed.

Significant predictors are indicated by asterisks (**p* < .05; ***p* < .01; ****p* < .001).

## Supplementary Figures


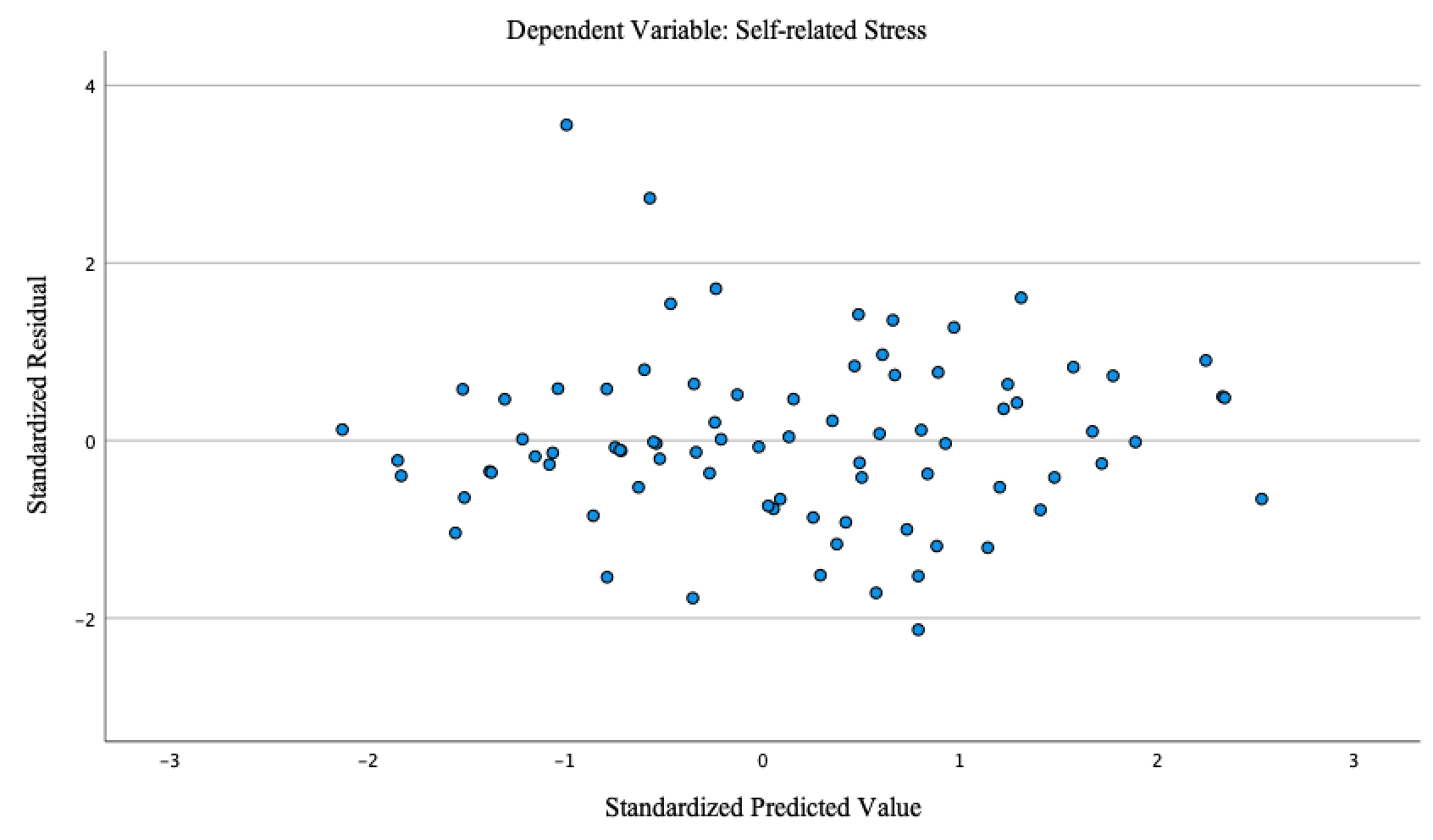


**Supplementary Figure 1.** Scatterplot of standardized residuals versus standardized predicted values for the clinical sample (dependent variable: self-related stress). Residuals for all models were inspected and showed comparable patterns; representative results are presented for the model with the highest *R^2^*in the clinical sample. Most standardized residuals were within the expected range (approximately ±2). One case had a relatively high residual of 3.5 (Cook’s distance = 0.13), and another case had a residual of 2.7 (Cook’s distance = 0.06); both cases did not unduly influence the regression results.


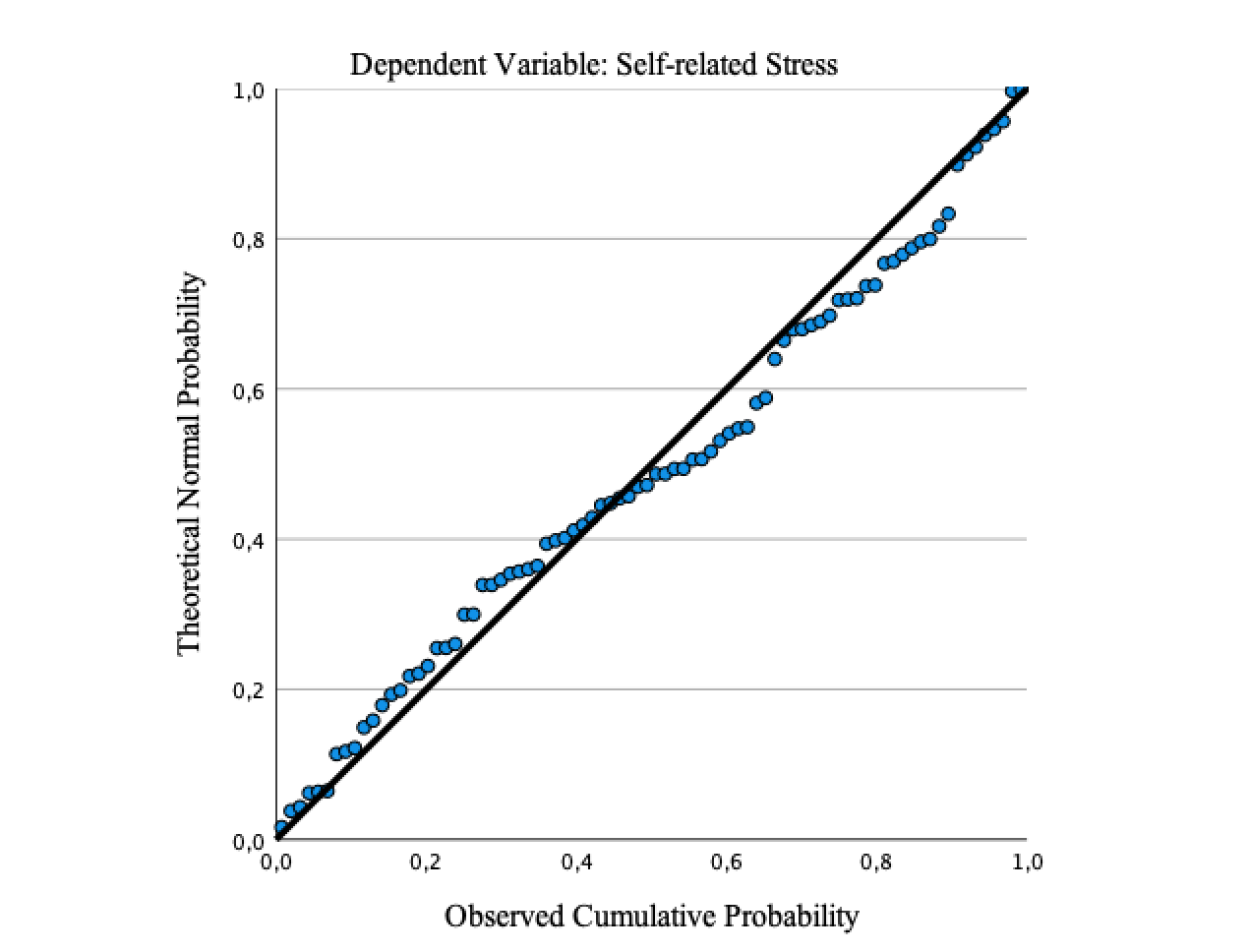


**Supplementary Figure 2.** Normal P–P plot of standardized residuals for the clinical sample (dependent variable: self-related stress). Residuals for all models were inspected and showed similar patterns; representative results are presented for the model with the highest *R^2^* in the clinical sample. Points lie close to the diagonal line, indicating that the residuals are approximately normally distributed.

**
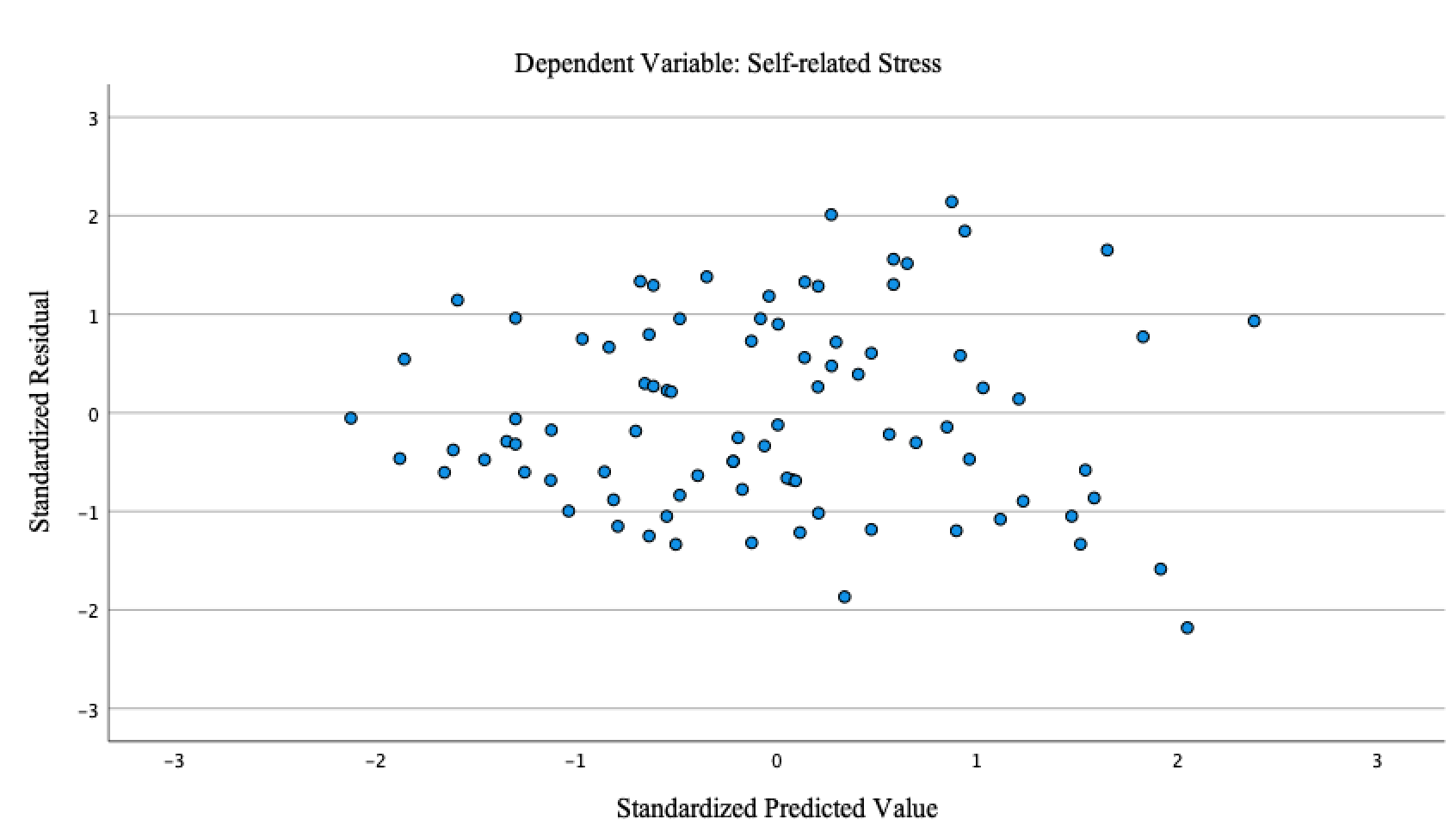
**

**Supplementary Figure 3.** Scatterplot of standardized residuals versus standardized predicted values for the non-clinical sample (dependent variable: self-related stress). Residuals for all models were inspected and showed comparable patterns; representative results are presented for the model with the highest *R^2^*in the non-clinical sample. Most standardized residuals were within the expected range (approximately ±2). Cook’s distance was also examined, and no cases unduly influenced the regression results.


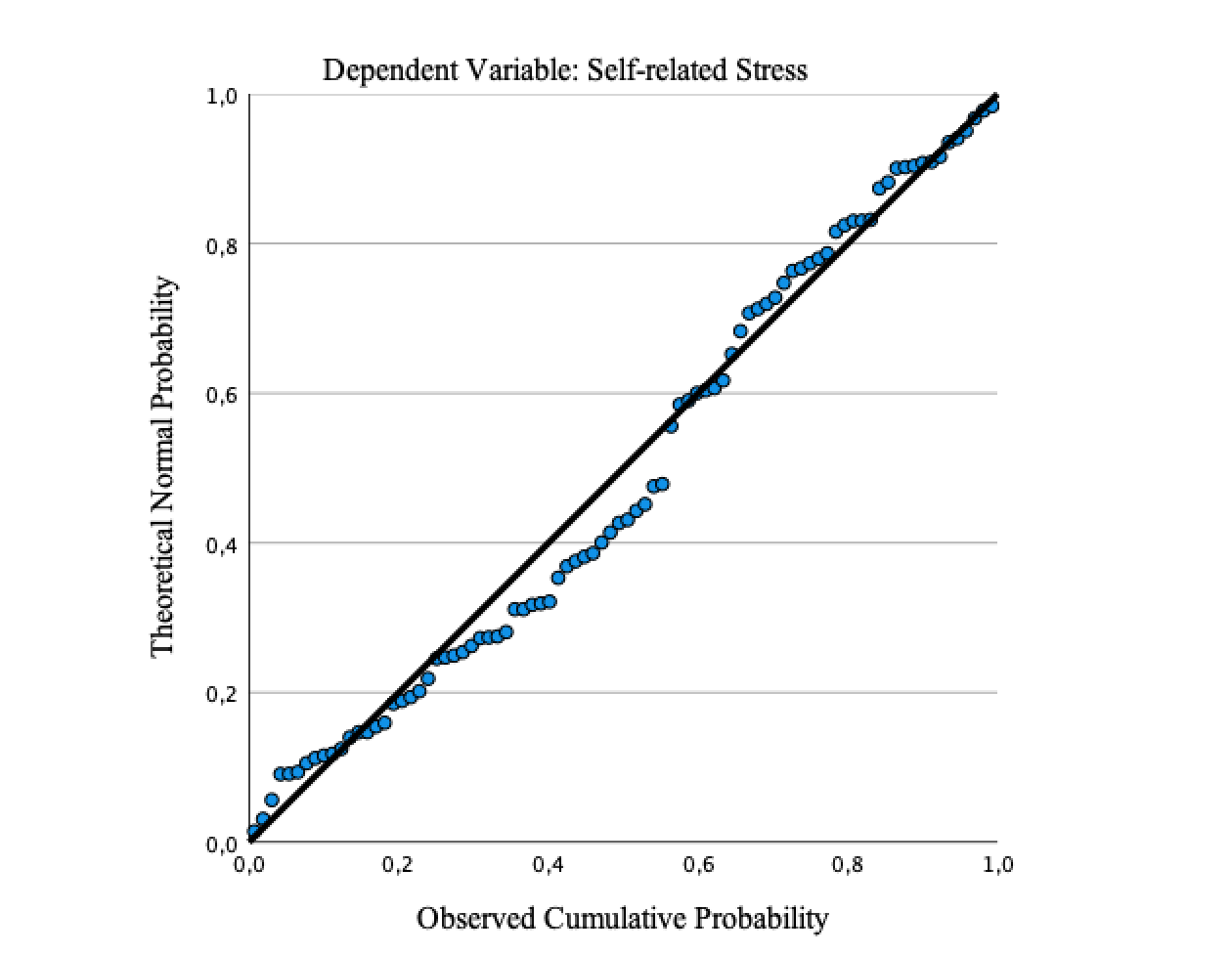


**Supplementary Figure 4.** Normal P–P plot of standardized residuals for the non-clinical sample (dependent variable: self-related stress). Residuals for all models were inspected and showed similar patterns; representative results are presented for the model with the highest *R^2^* in the non-clinical sample. Points lie close to the diagonal line, indicating that the residuals are approximately normally distributed.
